# A non-controlled, single arm, open label, phase II study of intravenous and intratumoral administration of ParvOryx in patients with metastatic, inoperable pancreatic cancer: ParvOryx02 protocol

**DOI:** 10.1186/s12885-017-3604-y

**Published:** 2017-08-29

**Authors:** Jacek Hajda, Monika Lehmann, Ottheinz Krebs, Meinhard Kieser, Karsten Geletneky, Dirk Jäger, Michael Dahm, Bernard Huber, Tilman Schöning, Oliver Sedlaczek, Albrecht Stenzinger, Niels Halama, Volker Daniel, Barbara Leuchs, Assia Angelova, Jean Rommelaere, Christine E. Engeland, Christoph Springfeld, Guy Ungerechts

**Affiliations:** 1 Coordination Centre for Clinical Trials, University Hospital Heidelberg, Marsilius-Arkaden, Tower West, Im Neuenheimer Feld 130.3, 69120 Heidelberg, Germany; 2 Oryx GmbH & Co KG, Marktplatz 1, 85598 Baldham, Germany; 3 Institute of Medical Biometry and Informatics, University Hospital Heidelberg, Marsilius-Arkaden, Tower West, Im Neuenheimer Feld 130.3, 69120 Heidelberg, Germany; 4 Department of Neurosurgery, Klinikum Darmstadt, Grafenstraße 9, 64283 Darmstadt, Germany; 5 Department of Medical Oncology, National Center for Tumor Diseases (NCT), Im Neuenheimer Feld 460, 69120 Heidelberg, Germany; 6 Central Pharmacy, University Hospital Heidelberg, Im Neuenheimer Feld 670, 69120 Heidelberg, Germany; 7 Department of Radiology, German Cancer Research Center, Im Neuenheimer Feld 280, 69120 Heidelberg, Germany; 8 Department of Pathology, University Hospital Heidelberg, Im Neuenheimer Feld 224, 69120 Heidelberg, Germany; 9 Tissue Imaging & Analysis Center (TIGA), University Heidelberg – BioQuant, Im Neuenheimer Feld 267, 69120 Heidelberg, Germany; 10 Institute of Immunology, Transplantation Immunology, University Hospital Heidelberg, Im Neuenheimer Feld 305, 69120 Heidelberg, Germany; 11 Department of Applied Tumor Virology, German Cancer Research Center, Im Neuenheimer Feld 242, 69120 Heidelberg, Germany

**Keywords:** H-1 parvovirus, Parvovirus, Oncolytic virotherapy, Pancreatic cancer, Pancreatic ductal adenocarcinoma, PDAC, Clinical protocol

## Abstract

**Background:**

Metastatic pancreatic cancer has a dismal prognosis, with a mean six-month progression-free survival of approximately 50% and a median survival of about 11 months. Despite intensive research, only slight improvements of clinical outcome could be achieved over the last decades. Hence, new and innovative therapeutic strategies are urgently required. ParvOryx is a drug product containing native parvovirus H-1 (H-1PV). Since H-1PV was shown to exert pronounced anti-neoplastic effects in pre-clinical models of pancreatic cancer, the drug appears to be a promising candidate for treatment of this malignancy.

**Methods:**

ParvOryx02 is a non-controlled, single arm, open label, dose-escalating, single center trial. In total seven patients with pancreatic cancer showing at least one hepatic metastasis are to be treated with escalating doses of ParvOryx according to the following schedule: i) 40% of the total dose infused intravenously in equal fractions on four consecutive days, ii) 60% of the total dose injected on a single occasion directly into the hepatic metastasis at varying intervals after intravenous infusions. The main eligibility criteria are: age ≥ 18 years, disease progression despite first-line chemotherapy, and at least one hepatic metastasis. Since it is the second trial within the drug development program, the study primarily explores safety and tolerability after further dose escalation of ParvOryx. The secondary objectives are related to the evaluation of certain aspects of anti-tumor activity and clinical efficacy of the drug.

**Discussion:**

This trial strongly contributes to the clinical development program of ParvOryx. The individual hazards for patients included in the current study and the environmental risks are addressed and counteracted adequately. Besides information on safety and tolerability of the treatment after further dose escalation, thorough evaluations of pharmacokinetics and intratumoral spread as well as proof-of-concept (PoC) in pancreatic cancer will be gained in the course of the trial.

**Trial registration:**

ClinicalTrials.gov-ID: NCT02653313, Registration date: Dec. 4th, 2015.

## Background

According to epidemiological estimations for 40 European countries the overall incidence of pancreatic cancer in the year 2012 amounted to approximately 10.5 cases per 100,000 inhabitants [1]. The figures for mortality were only slightly lower with 10.1 cases per 100,000, indicating the limited treatment options for this disease [1, 2]. Unlike in other neoplasms, the apparent mortality from pancreatic cancer has increased gradually in the past decades and was approximately 20 to 30% higher in 2014 than in 1970. This is probably due to an improvement of diagnostic procedures with a parallel increase in the number of properly documented disease cases. Nevertheless, pancreatic cancer is the only major cancer showing nearly no improvement of therapeutic outcome over the last decades [1–3].

Currently, there are no modalities for early diagnosis or screening for pancreatic cancer so that the disease is typically discovered only at advanced stages. Based on the analysis of the US National Cancer Database (NCDB) performed by the American Joint Committee on Cancer (AJCC) for the period between 1992 and 1998, the following relative distribution of disease stages at the time of the initial diagnosis can be assumed: stage I 9.8, stage II 21.9, stage III: 13.0, and stage IV 55.2%. The corresponding 5-year survival rates are: 25.3, 11.6, 2.7, and 0.7%, respectively [4]. The locally advanced (stage III) and metastatic disease (stage IV) are primarily not eligible for surgical intervention and therefore associated with poor prognosis. The current standard of care for these tumor stages relies upon different chemotherapeutic regimens.

Based on the results of a randomized, controlled clinical trial comparing the therapeutic efficacy of a combination of oxaliplatin, leucovorin, irinotecan, and 5-FU (FOLFIRINOX) to monotherapy with gemcitabine, FOLFIRINOX has been established as the first-line therapy in patients with inoperable pancreatic cancer who are in good physical condition([5]). Another phase III randomized, controlled clinical trial including 861 patients compared the clinical outcome after treatment with a combination of nab-paclitaxel and gemcitabine to gemcitabine alone [6]. As the drug combination showed significant increase in overall survival with acceptable toxicity, it was approved for the first-line treatment of inoperable disease by the US Food and Drug Administration (FDA) [7]. However, neither FOLFIRINOX nor the combination of nab-paclitaxel and gemcitabine bring about any relevant advantage in terms of long-term clinical outcome [5, 6].

## Trial rationale/justification

As briefly outlined above, despite intense efforts to improve treatment, the prognosis for pancreatic cancer patients is still disappointing. Therefore, all agents showing anti-tumor effects with an acceptable safety profile should undergo rapid clinical development to assess their therapeutic potential.

ParvOryx is a drug that contains parvovirus H-1 (H-1PV) as active substance. H-1PV is a small, single-stranded rodent DNA virus. The natural host is rat, but like other related parvoviruses, H-1PV is able to infect and replicate in cells of various other species including humans. Parvoviruses exert cytopathic effects mainly in neoplastic cells: they preferentially kill in-vitro-transformed and tumor-derived human and rodent cell lines, with limited-to-no cytocidal action in non-transformed cells [8]. Moreover, these viruses have been shown to have oncosuppressive properties, inhibiting the formation of spontaneous as well as chemically or virally induced tumors in laboratory animals [8, 9]. Furthermore, implants of tumor cells, including human neoplastic cells, were shown to be targets for parvoviral anti-cancer activity (oncolysis) in recipient animals [8–12]. Parvoviral cytotoxicity seems to be attributed to the viral nonstructural protein NS-1 [13].

H-1PV showed efficacy in preclinical, in-vitro models of pancreatic cancer. All investigated human pancreatic cancer cell lines, both of primary tumor and of metastatic origin, were susceptible to the stand-alone treatment with H-1PV, although to a varying extent [14, 15]. Synergistic increase of efficacy could be achieved by combination with valproic acid (VPA), a histone deacetylase inhibitor (HDACI) [14]. Moreover, based on the results from investigations on cellular pathways affected by H-1PV as well as by gemcitabine, synergistic effects of concomitant treatment with both agents can be anticipated [15]. Consecutive preclinical in-vivo investigations in animal models of pancreatic cancer carried out in mice and rats showed promising effects of H-1PV in the dose range between 1E09 and 2.5E09 plaque forming units (pfu). The anti-tumor effects were dose-dependent and the viral proteins were selectively expressed in the tumor as opposed to normal tissues. H-1PV virotherapy in an orthotopic pancreatic carcinoma model led to a significant delay in tumor growth and prolongation of survival, with 20% of the treated animals remaining disease-free for 16 weeks [15]. Importantly, in some cases, complete remission of pre-existing tumors was observed. Moreover, inoculation of the primary tumor with H-1PV at early stages of tumor development resulted in almost 50% suppression of distant metastases involving the visceral lymph nodes of the upper abdominal cavity and liver [15]. Also in animal models, the co-administration of VPA increased the potency of H-1PV, allowing a dose reduction by one power of ten down to 2.5E08 pfu without loss of efficacy [14].

Based on the findings described above, ParvOryx can reasonably be assumed to show efficacy against pancreatic cancer in humans. Ideally, the drug would not only be directly cytotoxic to the neoplastic cells but also induce anti-cancer vaccination by destruction of cancer cells and activation of the adaptive immune system. Based on the preclinical investigations synergistic effects with gemcitabine, a drug commonly used for treatment of pancreatic cancer, can be assumed. Thus, there is a strong rationale for treating patients suffering from pancreatic cancer with a combination regimen of ParvOryx and gemcitabine.

## Design and methods

### Aim

The trial aims at investigation of safety and tolerability, virus distribution and shedding as well as at evaluation of anti-tumor activity and clinical efficacy after multiple intravenous and a single intrametastatic administration of ParvOryx to patients suffering from pancreatic cancer.

### Objectives

#### Primary objectives

The primary objectives of the trial are related to the safety and tolerability of the Investigational Medicinal Product (IMP):

- Safety and tolerability assessed on the basis of physical examinations, chosen laboratory parameters, 12-lead electrocardiogram (ECGs), adverse events (AEs), and serious adverse events (SAEs),
- Assessment of humoral immune response to H-1PV after intravenous infusions and intrametastatic injection (detection of anti-drug-antibodies (ADA)),
- Investigation of the kinetics of H-1PV genomes in blood following intravenous and intrametastatic administration of the IMP by means of quantitative real-time polymerase chain reaction (qPCR),
- Investigation of virus shedding in faeces, urine, and saliva following intravenous and intrametastatic administration of the IMP.

#### Secondary objectives

The secondary objectives of the study are related to the anti-tumor activity and clinical efficacy of the IMP:

- Investigation of anti-tumor effects of ParvOryx by means of the following histo-immuno-pathological findings: i) extent of metastatis necrosis, proliferation rate and other pathological characteristics, ii) density of tumor-infiltrating immune cells,
- Quantity of cytokines and chemokines in tumor tissue,
- Investigation of viral replication in the tumor tissue by means of NS-1 detection in the tumor material,
- Investigation of the cellular immune response against viral proteins and tumor antigens by means of enzyme-linked immunospot assay (ELISPOT) and fluorescence-activated cell sorting (FACS),
- Progression-free survival (PFS) up to 6 months after the first administration of the IMP (determined by RECIST criteria),
- Morphological changes of the liver metastasis assessed by ultrasonography,
- Overall survival (OS) up to 6 months after the first administration of the IMP,
- Course of the tumor marker carbohydrate antigen 19-9 (CA 19-9) up to 6 months after the first administration of the IMP.

### Design

ParvOryx02 is a non-controlled, single arm, open label, dose-escalating, single center trial.

Due to an exploratory approach with regard to safety and tolerability of the IMP, no positive control is used. In face of the small size of the trial population and the intensity of collecting the biological samples, including multiple liver biopsies, no negative control (placebo) was implemented.

In a foregoing, first-in-man trial, referred to as ParvOryx01, comprehensive information on safety and tolerability of the IMP up to the total dose of 5E09 pfu administered by systemic (intravenous) as well as by local (intratumoral and intracerebral) route was obtained. Since the current trial includes further dose escalation up to the total dose of 1E10 pfu, a sequential design, including intervals of at least 28 days between treatments of consecutive subjects, is employed.

Due to the complex handling and administration of the IMP this trial is being performed in a single center with a sound experience in clinical research as well as in clinical management of patients with pancreatic cancer.

### Eligibility

Histologically confirmed pancreatic ductal adenocarcinoma with at least one hepatic metastasis is a prerequisite for inclusion in this trial. Moreover, patients have to fulfill the following main inclusion criteria: i) at least 18 years of age, ii) disease progression despite first-line therapy, iii) ECOG performance scale 0 or 1, iv) adequate main organ function, including normal thyroid function, v) negative beta-HCG-test and willingness to abide by the rules of adequate contraception.

Main criteria for exclusion of patients are: i) eligibility for surgery, ii) symptomatic cerebral, pulmonary, osseous metastases and/or peritoneal carcinomatosis, (iii) liver cirrhosis, previous splenectomy and/or severe respiratory impairment, (iv) chemo- and/or radiotherapy within 2 and 6 weeks prior to trial inclusion, respectively, (v) known allergy to iodinated contrast media, (vi) presumed contact to pregnant women and/or infants within 2 months after the first administration of the IMP.

### Sample size

As no confirmatory hypothesis tests are performed in this trial, the choice of sample size was not based on formal sample size calculation but on the following pragmatic considerations. The trial aims at evaluating safety and tolerability as well as proof-of-concept (PoC) regarding efficacy of ParvOryx in the treatment of pancreatic cancer. The number of seven subjects is assumed to be adequate to gain information on safety and tolerability of ParvOryx at the scheduled dose levels as required for continuation of the clinical drug development. Moreover, since PoC is mainly related to pathological and immunological parameters, the size of the trial population seems to be sufficient.

### Course of the trial

**Fig. 1:**
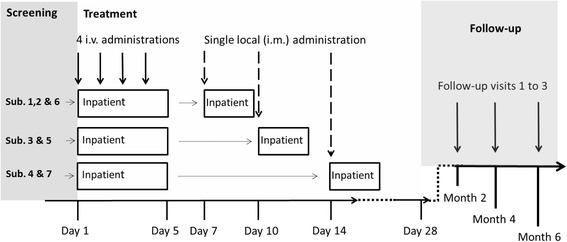
General overview of the course of the trial. The trial consists of three phases: *Screening,* aiming at verification of patients’ eligibility for the trial; *Treatment*, in which the IMP is administered and the chosen parameters on safety, tolerability, distribution and biological activity of ParvOryx are investigated; *Follow-up,* aiming at the long-term assessment of safety, tolerability, biological activity and clinical efficacy of ParvOryx. Abbreviations: i.m.: intrametastatic, i.v.: intravenous, Sub: subjects

**Fig. 2:**
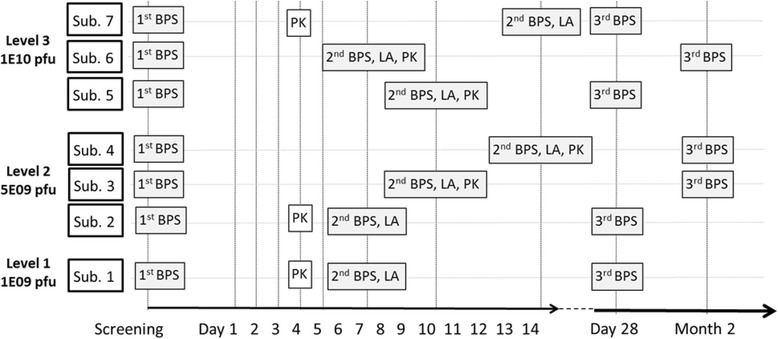
Schedule of the main trial-specific interventions. In order to account for potential time-dependent effects, the trial-specific interventions are to be carried out at different time points. Abbreviations: BPS: biopsy of liver metastasis, LA: local (intrametastatic) administration of ParvOryx, PK: thorough pharmacokinetic investigations, Sub: subject

A schematic overview of the trial is given in Fig. 1. For each individual subject, the trial consists of three phases, i.e. screening, treatment (including observation until study Day 28) and follow-up phase between study Day 28 and 6 months:

- *Screening*
  Screening of patients, aiming at assessment of their eligibility for the trial and collection of the baseline parameters, must be carried out within 2 weeks prior to the study inclusion. Potentially eligible patients are provided with comprehensive written and verbal information. Procedures that are carried out during the screening include:
  Written informed consent, demography and medical history, concomitant medication, physical examination, vital signs and 12-lead ECG, clinical chemistry, hematology and coagulation, CA19-9, ELISPOT and FACS, H-1PV-specific antibodies, serology of human immunodeficiency virus (HIV), hepatitis B virus (HBV) and hepatitis C virus (HCV), pregnancy test, thoracic computed tomography (CT) and abdominal magnetic resonance imaging (MRI), abdominal ultrasonography.
  At the end of the screening phase the inclusion and exclusion criteria will be reviewed and the final judgment on the subject’s eligibility will be made. If eligible, the subject will enter the treatment phase and receive the study-specific intervention.
- *Treatment (main intervention)*
  The IMP in this trial is ParvOryx, i.e. a GMP-grade preparation of H-1PV. The administration of the IMP is carried out as follows: i) 40% of the total dose, divided into four equal fractions (10% of the total dose each) infused intravenously (i.v.) over 2 h on four consecutive days, ii) 60% of the total dose injected on a single occasion directly into the hepatic metastasis under ultrasound guidance. The timing of intrametastatic injection differs between the trial subjects. The injection is to be performed either 6 or 9 or 13 days after the first i.v. administration of the IMP (see Fig. 1). The total doses of ParvOryx are: 1E09 pfu in the first subject, 5E09 pfu in three further subjects and 1E10 pfu in the last three subjects (see Table 1). The dose escalation and maintenance at the given dose level is only allowed if ParvOryx proved safe and well-tolerated in the previously treated subjects.
  The different time points for the intrametastatic injection were chosen to explore the most appropriate schedule for boosting the anticipated anti-tumor immune reaction. Furthermore, the tissue samples taken in parallel to the intrametastatic treatment will allow for assessment of pharmacokinetics and pharmacodynamics at the respective time points.
  There are two chemotherapeutics defined as non-investigational medicinal products (NIMP) in this trial: i) Gemcitabine, administered at the dose of 1000 mg/m^2^ of body surface area (BSA) on days 1, 8 and 15 of each 28-day cycle. The administration (cycle 1, day 1) is to be commenced 27 days after the first intravenous administration of ParvOryx, ii) Nab-paclitaxel, administered at the dose of 125 mg/m^2^ of BSA, given on days 1, 8 and 15 of each 28-day cycle, immediately prior to gemcitabine. Nab-paclitaxel is to be introduced only in case of disease progression despite previous treatment with ParvOryx and gemcitabine. Nab-paclitaxel has emerged as a second-line treatment option in PDAC after FOLFIRINOX failure [16]. Mechanistically, preclinical data suggest that nab-paclitaxel increases gemcitabine levels by decreasing intratumoral cytidine deaminase activity [17]. In absence of preclinical data of combining ParvOryx, gemcitabine and nab-paclitaxel, a triple therapy was not feasible within this trial. Nevertheless, this treatment option should be made available to patients with disease progression.
  Although the primary objective of this trial refers to the safety and tolerability of ParvOryx, the investigations related to the local anti-tumor activity and to the pharmacokinetics (PK) of H-1PV genomes are of substantial importance. In order to detect possible time-dependent differences, study days appointed for biopsies and PK sampling differ interindividually (see Fig. 2). Likewise, the thorough blood PK evaluations (PK profiles) are scheduled for different study days, i.e. they are performed either on the last day of i.v. administration (3 subjects) or on the day of intrametastatic administration (the remaining four subjects) of ParvOryx. In either case the timing of blood collection is as follows: The first sample is taken prior to the dosing of the IMP, the second sample up to 10 min after the end of the administration procedure, i.e. either at the end-of-infusion or after intrametastatic injection is completed; further samples are obtained 0.5, 1, 2, 4, 7 and 22 h thereafter. Three biopsies per subject are to be collected: i) prior to the overall first administration of the IMP, ii) 6, 9, or 13 days after the first i.v. administration of ParvOryx, directly prior to the intrametastatic administration, iii) either one or 2 months after the first i.v. administration of ParvOryx. Since cycle 1 of gemcitabine begins on day 27, effects of chemotherapy will be accounted for when interpreting biopsies taken 2 months after i.v. ParvOryx.

**Table 1:** Dosing of ParvOryx in the current trial

Total dose	Study Time	Individual dose and route of administration	Duration
Dose level 1 (1 subject)			
1E09 pfu	Day 1–4	1E08 pfu, intravenous infusion	2 h
	Day 7	6E08 pfu, intrametastatic injection	As slowly as feasible
Dose level 2 (3 subjects)			
5E09 pfu	Day 1–4	5E08 pfu, intravenous infusion	2 h
	Day 7 or 10 or 14	3E09 pfu, intrametastatic injection	As slowly as feasible
Dose level 3 (3 subjects)			
1E10 pfu	Day 1–4	1E09 pfu, intravenous infusion	2 h
	Day 7 or 10 or 14	6E09 pfu, intrametastatic injection	As slowly as feasible

- *Follow-up*
  During the follow-up phase, which extends up to 6 months after the first administration of the IMP, delayed and/or long-term effects of ParvOryx are evaluated. If no complications occur, the subjects are to attend the study visits at months 2, 4 and 6. At each visit safety, tolerability and clinical efficacy will be assessed by the parameters described above.
- *Trial schedule and duration*
  ParvOryx02 is the overall second clinical trial with the IMP ParvOryx. In this trial further dose escalation is planned. Since the starting dose level equals to the concluding dose level of the previous trial (ParvOryx01), a sequential escalation design is used. The subsequent patient may only receive the first dose of ParvOryx if the treatment proved safe and well-tolerated in the previous subject, i.e. if none of the following pre-defined events occurred up to 27 days after the first i.v. administration: i) elevation of alanine aminotransferase (ALAT), aspartate aminotransferase (ASAT), alkaline phosphatase (AP), bilirubin or c-reactive protein (CRP) > 3 times the baseline ii) neutrophil count <1.0 × 1E09/L or >12E09/L, iii) hemoglobin <7.5 g/L, iv) platelet count <5E10/L, v) INR > 2.5, aPTT >50 s., vi) occurrence of neurological symptoms with no other explanation than the administration of the IMP, vii) occurrence of thromboembolic event(s), vii) serious adverse events (SAE(s)) classified as at least ‘possibly’ related to the IMP viii) deteriorations in medical monitoring parameters (laboratory values, ECG, etc.), classified as at least ‘possibly’ related to the IMP and requiring countermeasures to avert conditions fulfilling at least one of the ‘seriousness’-criteria, ix) medical necessity to interrupt or to prematurely terminate the scheduled treatment. If any of the before mentioned events occur, an independent data safety monitoring board (DSMB) will be provided with all required data and consulted regarding the trial continuation and/or implementation of any modifications.
  The overall duration of the clinical trial, including completion of all follow-up visits related to the efficacy of the IMP in all subjects, is scheduled to last approximately 12 months.

### Specific hygienic measures

ParvOryx contains the active, replication-competent virus. Therefore, environmental safety has to be considered as an important factor in the context of administering ParvOryx.

Based on the findings from the previous clinical trial, the risk of virus transmission from study patients to other persons is very low even after the planned dose escalation, if the general hygienic measures are observed. Thus, there is no need for isolation of the patients treated with ParvOryx. However, to discover and appropriately meet the very unlike case of uncontrolled viral replication after administration of ParvOryx, certain measures have been implemented. The in-patient stay is to be continued until first occurrence of H-1PV-specific antibodies in serum or until all shedding samples (feces, urine, and saliva) are tested negative for H-1PV genomes. During each follow-up visit the presence of specific antibodies will be determined. If the antibodies fall below the detection limit, the subject has to be re-admitted to the trial unit and the extent of virus shedding is to be determined. If no viral genomes are shed in any matrix, no further measures are required and the subject may be discharged. Otherwise the subject remains in-patient until re-occurrence of H-1PV-specific antibodies or until all shedding samples are tested negative for viral genomes.

The only a-priori planned safety measure related to environmental safety is that subjects should entirely avoid contact with pregnant women and infants up to 2 months after the first administration of ParvOryx.

### Benefit/risk assessment

As discussed above, no satisfying therapeutic options exist for treatment of locally advanced and metastatic pancreatic cancer. The prognosis is dismal with a six-month progression-free survival of approximately 50% and a median survival of about 11 months [5].

The first open, non-randomized clinical trial with ParvOryx, referred to as ParvOryx01, evaluated safety and tolerability as well as antitumor activity and clinical efficacy in patients with progressive primary or recurrent glioblastoma multiforme (GBM). ParvOryx01 was completed in May 2015 [18]. The investigated doses ranged between 1E06 and 5E09 pfu. They were administered as combination of either multiple intravenous infusions or a single intratumoral injection and multifocal intracerebral injections at the end of tumor resection surgery. As in the current trial, the intravenous dose was divided into equal fractions which were given on consecutive days. The interval between the first administration and surgery with subsequent intracerebral injections of the drug was 10 days. In general, ParvOryx was safe and well-tolerated with only one potential serious adverse reaction observed after a combination of direct glioblastoma administration and intracerebral injection at the end of surgery. The clinical symptoms of the above reaction (mainly hydrocephalus and reduced level of consciousness) were strictly confined to the central nervous system, i.e. there was no causal link to the systemically available virus. As the potential underlying pathomechanisms remained unclear, the causal relationship to ParvOryx can neither be confirmed nor excluded. Of note, no comparable clinical events occurred in any of the other patients treated by the same route and with the same dose of ParvOryx. Since neither of the above routes of administration is used in the current study, there is no risk of similar adverse reactions.

The single intravenous dose at the initial level equals to that investigated at the highest level in ParvOryx01. Since the intravenous administrations are to be performed on four, instead of five consecutive days, the total intravenous dose is reduced to 80% of the dose investigated previously. The starting local, i.e. intrametastatic dose is more than four times lower than the highest doses injected intratumorally and intracerebrally in the foregoing trial. Since the metastatic/hepatic tissue is presumably far less susceptible to any kind of injury than the neuronal tissue of the brain, the chosen approach appears acceptable. Owing to the overall higher dose range investigated in the current study as well as to the planned co-treatment with gemcitabine, the steps of dose-escalation were chosen more conservatively than in the previous trial.

As in the foregoing trial, the treatment of a consecutive subject is only allowed if the treatment was well-tolerated in the previous subject who, as per protocol, underwent a close medical monitoring during the treatment and up to 3 weeks thereafter. This sequential schedule of enrolment minimizes the individual risks for the included patients.

Although H-1PV is non-pathogenic in man, strong preventive hygienic measures were implemented in the ParvOryx01 study [18]. Amongst others, the investigation of virus distribution and excretion belonged to the main objectives of the trial. Considering the fact that no active virus could ever be detected in any body fluid (faeces, urine, and saliva) and taking into account the rapid formation of virus-specific antibodies, the transmission of H-1PV from the trial patients is considered as highly unlikely. Thus, it is well justifiable to omit the strict isolation conditions applied previously and to rely on the general hygienic standards which are routinely applied at the trial center. In case of a surface contamination with ParvOryx, an adequate disinfection is to be carried out. Since H-1PV was shown to have some embryo- and fetotoxic effects in rodents [19, 20], the trial subjects are obliged to strictly avoid contact with pregnant women and newborn infants for the period of 2 months after beginning of the treatment with ParvOryx. This is to be considered an additional safety measure, i.e. there are currently no indications for a predisposition of pregnant women or infants for an infection with H-1PV.

The risk of trial-specific procedural complications related to the intrametastatic administration of ParvOryx, collection of tissue and blood samples is generally very low.

Taken together, the current protocol of ParvOryx02 trial is well justifiable and may be associated with individual benefits for the included patients. Moreover, the trial will yield important information required for further clinical development of ParvOryx, which may have important implications for the general population of patients with pancreatic cancer. Taken together, the individual hazards for study subjects and the environmental risks are well predictable and acceptable.

### Statistical analysis

- *Analysis variables*
  - *Safety and tolerability will be assessed on the basis of the following parameters*:
    AEs and SAEs; physical examinations, vital signs, 12-lead ECGs, and chosen laboratory parameters (clinical chemistry, hematology, coagulation); viremia and virus shedding (H-1PV genomes (Vg) and active virus (H-1PV) in body fluids); virus-specific antibodies.
  - *Efficacy will be assessed on the basis of the following parameters*:
    Investigation of the metastatic tissue: findings in general pathological examination, detection of H-1PV by FISH and qPCR, assessment of tumor infiltration with immune cells, determination of quantity and distribution of cytokines and chemokines, determination of H-1PV protein expression (NS-1).
    Parameters derived from blood: determination of absolute and relative abundance of distinct immune cell subsets as determined by FACS, investigation of cellular anti-viral and anti-tumor immunity by ELISPOT.
    Clinical Parameters: progression-free survival (PFS) and overall survival (OS) assessed by RECIST-criteria.
- *Statistical methods*
  - Safety analysis: AEs will be summarized by MedDRA system organ class and preferred term. Separate tabulations will be produced for all treatment-emergent AEs, treatment-related AEs (those considered by the Investigator as at least possibly IMP-related), SAEs, and discontinuations due to AEs. Summary tables and by-patient listings will be provided for AEs, SAEs, events leading to discontinuation of treatment, and deaths. Summary tables and by-patient listings will be provided for clinical laboratory data and vital signs data, presented as both actual values and changes from baseline relative to each on-study evaluation. Details of any abnormalities will be included in patient listings.
  - Efficacy analysis: No confirmatory statistical analyses will be performed. All recorded variables (see above) will be analyzed descriptively by providing by-patient listings as well as calculating appropriate summary measures as mean, standard deviation, median, minimum and maximum or absolute and relative frequencies, respectively. If appropriate, changes from baseline relative to each on-study evaluation will be considered. For time-to-event endpoints (progression-free survival and overall survival), Kaplan-Meier estimates and summary measures of the survival function will be provided. Additionally, analyses will be performed separately for each particular dose level. The course of variables over time will be depicted for the total analysis population as well as for each subject.

## Discussion

Among other emerging biopharmaceuticals, the clinical use of oncolytic viruses appears to be a promising treatment option for various malignancies. Currently, there is a range of mainly genetically modified oncolytic viruses at different stages of clinical development [21–31]. Recently, talimogene laherparepvec (T-vec) received market authorization by the U.S. Food and Drug Administration (FDA) for treatment of melanoma patients with injectable but non-resectable skin and/or lymphatic lesions [32]. In general the tolerability of oncolytic viruses after systemic and/or local administration is very good with none or only mild unspecific adverse reactions such as fatigue, chills or slight fever. There are no indications for major organ toxicities, local tissue damage or induction of adverse immune effects. The oncolytic viruses are used either as monotherapies or in combination with established chemotherapeutics and/or targeted therapies. There are strong indications for anti-tumor activity and clinical efficacy in connection with either approach. However, in most cases the optimum mode of administration, including dosing schedule and type as well as timing of concomitant treatments still needs to be specified. Interestingly, concomitant therapy with oncolytic viruses and checkpoint inhibitors seems not to influence the safety and tolerability of either treatment [33]. This is of high relevance as the combination may enhance individual anti-tumor immune responses with an improvement of clinical outcome.

Recently, ParvOryx was clinically investigated for its safety and tolerability, anti-tumor activity, immunological effects, and clinical efficacy in patients with GBM [18]. In this study the virus was administered intravenously and into the tumor or in the tumor bed directly after resection. The drug was safe and well-tolerated and showed a promising profile of anti-tumor effects and signs of clinical efficacy, i.e. prolonged survival. However, the optimum dose as well as the most appropriate route and schedule of administration have to be further investigated. The current, second trial with ParvOryx, addresses these questions. Since the total dose of the drug will be further escalated, the primary objective is to evaluate the safety and tolerability of the treatment. At the beginning of the trial, i.e. at the first level, the total dose of ParvOryx equals to the highest total dose in the previous trial. Since a relevant part of the total dose has to be administered directly into the liver metastasis, it is indicated to include one up-front patient at this level, in order to obtain first information on the local tolerability of the drug after direct injection in the liver. Considering the fact that in the foregoing study the local tolerability in the neuronal tissue was very good, no safety-related issues are expected in this context. Since the potential hazards for the consecutive patients have to be minimized as far as possible, a sequential, dose escalation design with extended intervals between enrollment of consecutive subjects and rather conservative dose escalation steps was chosen. As in the previous trial, a broad range of different investigational parameters was implemented. Apart from extended safety tests, various measurements enabling insights into the mode and extent of action of ParvOryx, including local virus availability in the tumor, triggering of changes at the tissue level and induction of virus- and PDAC-specific immune responses were included into the current protocol. Moreover, in order to account for presumable time-dependency of pharmacokinetic characteristics, virus disposition in the tumor tissue and related pharmacodynamics, varying intervals were intercalated between the intravenous administrations on the one hand and biopsies of liver metastases, thorough PK-profiles as well as local administrations of ParvOryx on the other hand.

The IMP contains an active, replication-competent parvovirus H-1PV. Although H-1PV is non-pathogenic in humans, biosafety is still considered a relevant issue in the context of administration of ParvOryx. Based on the results from the previous trial, a transmission of H-1PV from trial patients to others is highly unlikely, since general hygienic measures applicable to the handling of chemotherapeutics, consumables and nursing of patients are implemented. Owing to the pre-clinical findings showing an embryo- and fetotoxicity of H-1PV in rodents, patients’ contact with pregnant women and infants is restricted as an additional precaution.

In summary, the current trial will provide further crucial information within the clinical development program of ParvOryx. Since there were pronounced anti-tumor effects of the drug in various preclinical in-vitro and in-vivo models of pancreatic cancer, the trial will hopefully bring clinical benefits for study patients and, in consequence, for the general patient population.

## Abbreviations

(N)IMP: (Non-) Investigational Medicinal Product
(S)AE: (Serious) Adverse Event
ADA: Anti-drug Antibodies
AJCC: American Joint Committee on Cancer
ALAT: Alanine aminotransferase
aPTT: activated Partial Thromboplastin Time
ASAT: Aspartate aminotransferase
Beta-HCG: Beta-human Chorionic Gonadotropin
BSA: Body surface area
CA19–9: Carbohydrate antigen 19–9
CRP: C-reactive protein
CT: Computed tomography
DSMB: Data safety monitoring board
ECG: Electrocardiogram
ECOG: Eastern Cooperative Oncology Group
ELISPOT: Enzyme-linked immunospot assay
FACS: Fluorescence-activated cell sorting
FDA: US Food and Drug Administration
FISH: Fluorescence in-situ Hybridization
GBM: Glioblastoma multiforme
H-1PV: Parvovirus H-1
HDACI(s): Histone deacetylase inhibitor(s)
INR: International Normalized Ratio
MedDRA: Medical Dictionary for Regulatory Activities
NCDB: US National Cancer Database
NS-1: Non-structural Protein 1
OS: Overall survival
PFS: Progression-free survival
Pfu: Plaque forming units
PK: Pharmacokinetics
PoC: Proof-of-concept
qPCR: quantitative real-time polymerase chain reaction
RECIST: Response Evaluation Criteria in Solid Tumors
VPA: Valproic acid
